# Dose‐response relations and the moderators between different types of exercise and cognitive functions in older people with MCI: a secondary analysis of a three‐arm randomized controlled trial

**DOI:** 10.1002/alz70860_100700

**Published:** 2025-12-23

**Authors:** Xiuxiu Huang, Shifang Zhang, Xiaoyan Zhao, Qiaoqin Wan

**Affiliations:** ^1^ School of Nursing, Shanghai Jiao Tong University, Shanghai, Shanghai, China; ^2^ School of Nursing, Peking University, Beijing, Beijing, China

## Abstract

**Background:**

Identifying the dose‐response relations between exercise and cognition contributes to developing individualized exercise programs. This study aims to explore the dose‐response relations between different types of exercise and cognition, as well as the potential moderators.

**Method:**

This is a secondary analysis of a three‐arm randomized controlled trial. Older adults with mild cognitive impairment (MCI) were randomized to aerobic exercise (AE), resistance exercise (RE), or control group, with 36 individuals in each group. Remotely supervised AE and RE training were conducted separately. Exercise dose was recorded. Cognitive functions were assessed at pre‐ and post‐intervention.

**Result:**

Twenty‐nine participants in AE group and 33 in RE group completed interventions. The median exercise doses per week were 2.76 (1.92, 3.72) (hours) for AE, and 3.00 (2.78, 3.39) (relative strength*sessions) for RE. After deleting outliers, 28 participants in AE group and 30 in RE group were included to dose‐response analyses. Paired t‐tests confirmed the effects of AE and RE programs. Linear regressions revealed positive dose‐response relations between AE and global cognition (β = 0.76, 95%CI (0.00,1.52), *p* = 0.051), and between RE and processing speed (β = 5.32, 95%CI (2.07,8.56), *p* = 0.003). Age, body mass index, physical activity levels, and comorbidities were moderators of the relations between AE dose and cognitive responses, while age, gender, body mass index, and sleep duration were moderators of the association between RE dose and cognitive responses.

**Conclusion:**

Positive dose‐response relations exist between distinct types of exercise and cognition which may be moderated by some factors. This study provided references for developing personalized exercise programs to optimize intervention efficiency and maximize cognitive benefits.

